# Prevalence of Group B *Streptococcus* Colonization and Invasive Infection in Nigeria: A Systematic Review and Meta-Analysis

**DOI:** 10.3390/medsci14010093

**Published:** 2026-02-15

**Authors:** Abdulrakib Abdulrahim, Victor Abiola Adepoju, AbdulRahman Muthanna, Bashar Haruna Gulumbe, Mohd Hafis Yuswan, Mohd Nasir Mohd Desa, Syafinaz Amin-Nordin

**Affiliations:** 1 Department of Medical Microbiology, Faculty of Medicine and Health Sciences, Universiti Putra Malaysia, Serdang 43400, Malaysia; abdulrakib161@gmail.com; 2 Department of HIV and Infectious Diseases, Jhpiego Nigeria (An Affiliate of Johns Hopkins University), Abuja 900911, Nigeria; 3 Department of Biomedical Sciences, Faculty of Medicine and Health Sciences, Universiti Putra Malaysia, Serdang 43400, Malaysia; mansoor@upm.edu.my (A.M.); mnasir@upm.edu.my (M.N.M.D.); 4 Department of Microbiology, Faculty of Science, Federal University Birnin-Kebbi, Birnin Kebbi 860001, Nigeria; bashar.haruna@fubk.edu.ng; 5 Laboratory of Halal Services, Halal Products Research Institute, Universiti Putra Malaysia, Serdang 43400, Malaysia; hafisyuswan@upm.edu.my; 6 Hospital Sultan Abdul Aziz Shah, Universiti Putra Malaysia, Serdang 43400, Malaysia

**Keywords:** *Streptococcus agalactiae*, maternal carriage, invasive GBS infection, neonatal sepsis, Nigeria, systematic review, epidemiology

## Abstract

**Objective**: This study provides the first systematic synthesis of the burden of Group B *Streptococcus* (GBS) colonization and invasive disease in Nigeria, with emphasis on prevalence, serotypes, and sequence types (STs). **Method**: This systematic review and meta-analysis were conducted in accordance with the PRISMA guidelines and was registered on PROSPERO (CRD420251155310). Searches were conducted across multiple databases, including Scopus, ScienceDirect, Web of Science, PubMed, Dimensions, and African Journals Online, as well as in Google Scholar and Google to identify relevant articles. In total, 426 records were retrieved, of which 43 studies met the inclusion criteria. A random-effects model was applied to estimate the pooled prevalence. **Result**: The pooled prevalence of GBS colonization in Nigeria was 12.0% (95% CI: 9.0–15.0%). Higher colonization rates were observed in Southern Nigeria (13.0%) than in Northern Nigeria (9.0%). The neonatal colonization rate was 16.0%. Colonization rates were 13.0% in pregnant women and 8.0% in non-pregnant individuals. Human immunodeficiency virus status showed no significant association with GBS colonization among pregnant women (OR = 1.47, *p* = 0.17). Invasive GBS disease was uncommon (3.0%) and occurred only in neonates. Across included studies, serotypes V and II were the most frequently reported, with ST19, ST182, and ST28 being the predominant STs. **Conclusions**: GBS colonization is common in Nigeria, with marked regional variation and heightened neonatal vulnerability to invasive GBS infections. Notably, nineteen states lacked surveillance data, highlighting substantial gaps in national monitoring. These findings highlight the importance of strengthening prevention strategies, expanding surveillance coverage, and implementing maternal screening and immunization programs to mitigate the burden of GBS.

## 1. Introduction

*Streptococcus agalactiae* (Group B *Streptococcus*; GBS) is a Gram-positive bacterium that occupies a unique niche at the interface of commensalism and invasive disease [1]. It commonly colonizes the gastrointestinal and genitourinary tracts of humans without symptoms, yet under certain circumstances, it can cause severe infections [1,2]. The clinical impact of GBS varies across populations, ranging from neonatal sepsis, pneumonia, and meningitis to maternal puerperal infections, chorioamnionitis, and invasive disease in non-pregnant adults, particularly the elderly and immunocompromised [2,3,4,5,6]. A 2020 global estimate attributed nearly 20 million maternal colonizations to GBS, resulting in approximately 394,000 infant infections, 58,000–92,000 neonatal deaths, 37,000 cases of long-term disability, more than 40,000 maternal infections, and 46,000 stillbirths [2].

GBS is heterogeneous. Isolates are classified into ten capsular polysaccharide serotypes (Ia–IX) [7,8,9] and by sequence types (STs) using multilocus sequence typing (MLST) [9,10,11]. Serotype and lineage distributions vary by region, host population, and clinical syndrome [3,7,9,12]. Globally, serotypes Ia, III, and V are predominant, associated with ST23, ST17, and ST1, respectively [6,8,11]. These differences are critical as they influence vaccine design, disease risk, and interpretation of surveillance data [11].

Maternal colonization prevalence also varies widely [13]. Multicountry studies report rates of 10–30%, though with heterogeneity by country and sampling methods [7,14,15,16]. In sub-Saharan Africa, prevalence estimates remain fragmentary due to limited surveillance and laboratory capacity [8]. Local prevalence data are essential since maternal colonization is the primary upstream risk factor for neonatal exposure and early-onset disease (EOD), making colonization, serotype distribution, and ST data vital for informing intrapartum antibiotic prophylaxis (IAP) policies and maternal vaccine strategies [13,17,18].

Nigeria, home to over 221 million people and one of the world’s highest birth rates, records 34.3 neonatal deaths per 1000 live births, ranking fifth globally [19]. This population growth significantly impacts public health [20]. Despite this, GBS data remain fragmented, with variable colonization and invasive estimates and limited serotype/ST characterization [12,18,21,22,23,24]. Weak surveillance systems, uneven laboratory capacity, and limited intrapartum screening further constrain detection [7,12,14,15,16]. This study represents the first systematic review and meta-analysis of the burden of GBS in Nigeria. We aimed to synthesize evidence on colonization and invasive disease, determine pooled prevalence across populations, serotype and ST distributions, and identify knowledge gaps to inform prevention strategies.

## 2. Methods

### 2.1. Protocol Registration and Reporting Standards

The review protocol was registered with the International Prospective Register of Systematic Reviews (PROSPERO) on 30 September 2025 under the registration number CRD420251155310. This systematic review and meta-analysis were conducted in accordance with the PRISMA 2020 guidelines (Supplementary Table S1), focusing on the prevalence of GBS colonization and invasive disease among neonates, pregnant women, and non-pregnant populations in Nigeria [25].

### 2.2. Eligibility Criteria

Original studies that described the study population and design and reported the prevalence (or sufficient data to calculate prevalence) of GBS colonization or invasive disease among neonates, pregnant women or non-pregnant populations in Nigeria and/or study which conducted serotyping or MLST for GBS isolates were includes while exclusion criteria include studies of GBS in other hosts, human GBS studies with unclear results, reviews, letters, commentaries, editorials, and studies that did not provide proportion/prevalence data among human participants are excluded.

### 2.3. Information Sources

We systematically searched literature published between 1 January 1984, and 31 December 2024, in Scopus, ScienceDirect, Web of Science, PubMed, Dimensions, Africa Journals Online, Google Scholar, and Google.

### 2.4. Search Strategy

Search terms combined “*Streptococcus agalactiae*” OR “Group B *Streptococcus*” OR “Group B streptococci” OR “Group B streptococcal” AND “Nigeria”. Detailed search strings for each database are provided in Supplementary Table S2.

### 2.5. Selection Process

**Figure 1:**
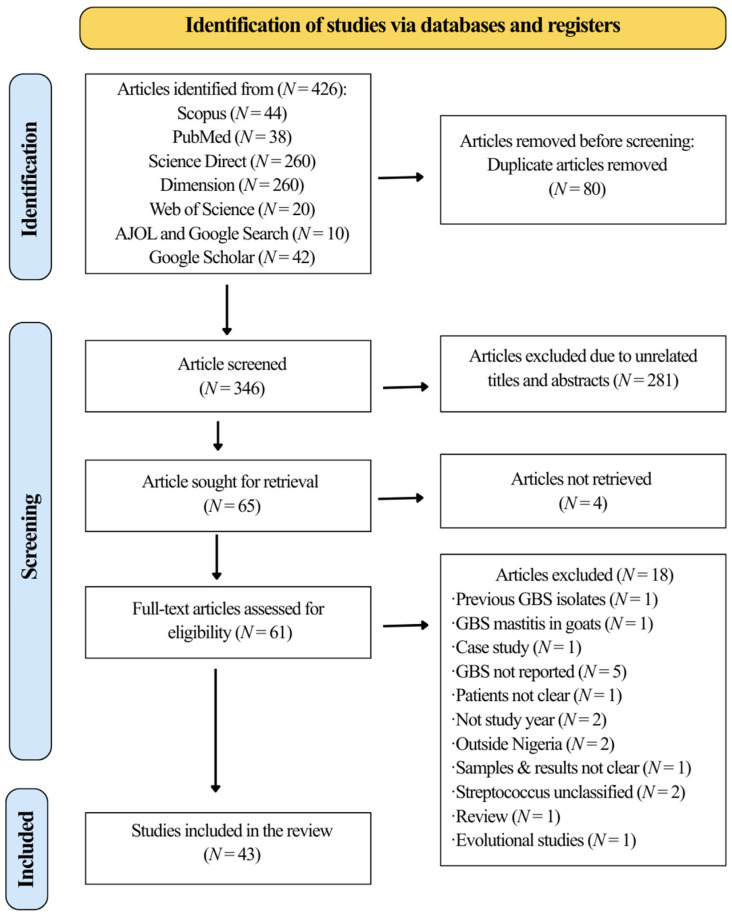
The study flow diagram demonstrates the study selection process.

All 426 records were imported into Rayyan software, and 80 duplicates were removed using a combination of exact matching criteria (title and author) and fuzzy logic [26]. After deduplication, 346 unique records remained. Two reviewers (AA and BHG) independently screened the titles, abstracts, and full texts of the articles. Of these, 281 articles were excluded at the title and abstract screening stage because they were not related to GBS in Nigeria. Sixty-five articles were sought for retrieval, of which four could not be retrieved. The remaining 61 articles underwent full-text screening, resulting in the exclusion of 19 studies. These excluded studies included review articles, letter to editors, commentaries, studies conducted in other countries, studies involving non-classified *Streptococcus* species, GBS studies in animals, non-GBS studies, studies with unclear sample sizes and results, and studies conducted outside the period 1984 to 2024 (Supplementary Table S3). Finally, 42 articles met the inclusion criteria and were included in the study. Any disagreements were resolved through discussion or, when necessary, by consultation with a third reviewer (VAA). The PRISMA 2020 flow diagram (Figure 1) illustrates the study selection process and the reasons for exclusion at the full-text stage.

### 2.6. Data Collection Process

Data were extracted into an Excel spreadsheet by two independent reviewers (AA and VAA) and cross-checked; disagreements were resolved by consensus. Extracted items included: first author (or first and second author where two authors were listed), publication year, state and geopolitical zone, study population (neonates, pregnant women, non-pregnant), sample size, number of GBS identified, specimen type, serotype distribution, and ST data (if performed) (Table 1).

### 2.7. Study Risk of Bias Assessment

AA and VAA independently assessed methodological quality using an adapted Joanna Briggs Institute (JBI) critical appraisal checklist [27]. A composite quality score (0–10) was assigned to each study using an adapted JBI checklist for prevalence studies. We prespecified ≥6/10 as moderate to high quality to reflect adequate sampling, an appropriate case definition, valid measurement, and acceptable response rates. Studies with scores < 6 were classified as lower quality (Supplementary Table S4). BHG adjudicated any disagreements.

### 2.8. Synthesis Methods

The data were synthesized using an online meta-analysis software available at https://metaanalysisonline.com (4 October 2025) [28]. In view of the substantial heterogeneity across studies, pooled prevalence estimates of GBS colonization and invasive infection in Nigeria were calculated using a random-effects model with 95% confidence intervals as well as meta-regression. The analyses were stratified by geographical region and by population groups, including neonates, pregnant women, and non-pregnant women. Serotype and ST distributions were summarized descriptively. We calculated pooled odds ratios and 95% confidence intervals. Results will be presented as tables and graphical displays, including forest plots and a map.

**Table 1:** Summary of the characteristics of the included studies.

S/N	Author	Year	State	Zone	Population	Specimen	Isolation	Sample Size	No. of GBS Isolates	Prevalence (%)	Serotype Identified	ST Identified
1.	Akadri et al. [21]	2019	Osun	SW	Pregnant women	Vaginal and rectal swabs	Colonizing	184	51	28	NR	NR
2.	Akinlolu et al. [29]	2018	Osun	SW	Pregnant women and neonates	Vaginal, rectal, and umbilical cord	Colonizing	340	98	29	NR	NR
3.	Akinniyi et al. [30]	2017	Kaduna	NW	Pregnant women	Vaginal and rectal swabs	Colonizing	220	19	9	NR	NR
4.	Anosike et al. [23]	2017	Cross river	SS	Pregnant and non-pregnant women	High vaginal swab	Colonizing	122	26	21	NR	NR
5.	Bamidele et al. [31]	2022	Ekiti	SW	Pregnant women and neonates	Vaginal and rectal swabs	Colonizing	252	45	18	NR	NR
6.	Bello et al. [22]	2016	Oyo	SW	Pregnant women	Vaginal and rectal swabs	Colonizing	240	23	10	NR	NR
7.	Biobaku et al. [32]	2017	Osun	SW	HIV (+ and −) pregnant women	Vaginal and rectal swabs	Colonizing	198	36	18	NR	NR
8.	Bob-Manuel et al. [33]	2021	Rivers	SS	Pregnant women	Vaginal and rectal swabs	Colonizing	185	35	19	Ia (10 isolates), Ib (1), II (8), III (8), IV (4), and V (15).	NR
9.	Bob-Manuel et al. [34]	2021	Rivers	SS	Pregnant women	Isolates from the previous study by Bob-Manuel et al. [33]						ST1 (1), ST2 (2), ST8 (1), ST17 (1), ST19 (9), ST23 (1), ST24 (4), ST26 (1), ST28 (3), ST182 (3), ST486 (5), ST1336 (2)
10.	Dozie-Nwakile et al. [35]	2022	Enugu	SE	Pregnant and non-pregnant women, neonates, couples with an infertility condition.	Vaginal, endocervical swabs, semen, urethral swabs, and neonate (not mentioned)	Colonizing	300	61	20	NR	NR
11.	Egbule et al. [36]	2024	Delta	SS	Pregnant women	Vaginal and rectal swabs	Colonizing	87	38	44	NR	NR
12.	Elikwu et al. [37]	2016	Lagos	SW	pregnant women and neonates	Vaginal and rectal	Colonizing	353	70	20	Ia (11 isolates), Ib (9), II (8), III (10), V (7), and VI (1).	NR
13.	Elikwu et al. [38]	2016	Lagos	SW	Pregnant women	Vaginal and rectal	Colonizing	300	59	20	NR	NR
14.	Ella et al. [39]	2008	Kaduna	NW	Neonate	Blood	Invasive	50	1	2	NR	NR
15.	Emembolu [40]	1989	Kaduna	NW	Infertile women	uterine curettage	Colonizing	114	10	9	NR	NR
16.	Ezeonu and Agbo [41]	2014	Enugu	SE	Pregnant and non-pregnant women	Vaginal and rectal swabs	Colonizing	400	47	12	NR	NR
17.	Gambo et al. [42]	2024	Sokoto	NW	Pregnant women	Vaginal and rectal swabs	Colonizing	152	7	5	NR	NR
18.	Hoppe et al. [43]	1986	Ogun	SW	Neonates	Pharyngeal aspirate, the nose, ear, and anus	Colonizing	200	38	19	Reported but not clearly grouped	NR
19.	Ibrahim et al. [44]	2021	Sokoto	NW	Pregnant women	Vaginal/rectal	Colonizing	185	7	4	NR	NR
20.	Idih et al. [45]	2019	Imo	SE	Pregnant women and neonates	Vaginal and rectal swabs	Colonizing	360	17	5	NR	NR
21.	Kwatra et al. [12]	2024	Abuja	NC	Mothers and neonates	Vaginal, urine, rectal swabs, umbilicus, outer ear, axillary fold, rectum, and throat	Colonizing	1759	405	23	Reported but not clearly grouped	NR
22.	Makinde et al. [18]	2022	Lagos	SW	HIV (+ and −) pregnant women	Vaginal and rectal swabs	Colonizing	244	8	3	NR	NR
23.	Medugu et al. [17]	2017	Abuja	NC	Mothers and neonates	Rectal and vaginal samples and the external auditory meatus for neonates	Colonizing	1000	266	27	V (112); II (60), Ia (34), III (29), IV (3), and NT (7)	ST19 (9), ST182 (7), ST1 (4), ST28 (3), ST17 (3), ST23 (3), ST2 (1), ST196 (1), ST8 (1), ST26 (1), ST24 (1), ST762 (1)
24.	Nanbol et al. [46]	2021	Plateau	NC	Pregnant and non-pregnant	Vaginal and rectal swabs	Colonizing	300	9	3	NR	NR
25.	Njoku et al. [47]	2018	Cross River	SS	HIV (+ and −) pregnant women	Vaginal and rectal swabs	Colonizing	168	18	11	NR	NR
26.	Nsagha et al. [48]	2000	Plateau	NC	Pregnant and non-pregnant women	endocervical and anorectal swabs	Colonizing	162	11	7	NR	NR
27.	Nsagha et al. [24]	2012	Plateau	NC	Non-pregnant women	Vaginal and rectal swabs	Colonizing	56	2	4	NR	NR
28.	Nwachukwu et al. [49]	2007	Cross River	SS	Pregnant women	Vaginal and rectal swabs	Colonizing	200	18	9	NR	NR
29.	Ojo et al. [50]	2019	Lagos	SW	Pregnant women	Vaginal and rectal swabs	Colonizing	140	6	4	NR	NR
30	Ojukwu et al. [51]	2006	Ebonyi	SE	Neonates	Blood	Invasive	138	1	1	NR	NR
31.	Okolo et al. [52]	1985	Edo	SS	Neonates	Blood	Invasive	177	1	1	NR	NR
32.	Okon et al. [53]	2013	Borno	NE	Pregnant women	Vaginal and rectal swabs	Colonizing	133	13	10	NR	NR
33.	Okon et al. [54]	2023	Akwa Ibom	SE	HIV (+) patients	Sputum	Colonizing	61	5	8	NR	NR
34.	Olanisebe and Adetosoye [55]	1986	Oyo	SW	Pregnant women	Vaginal and rectal swabs	Colonizing	500	8	2	NR	NR
35.	Onipede et al. [56]	2012	Osun	SW	Pregnant women	Vaginal and rectal swabs	Colonizing	150	17	11	NR	NR
36.	Onwuezobe and Effiom [57]	2016	Akwa Ibom	SE	Pregnant women	Vaginal and rectal swabs	Colonizing	150	2	1	NR	NR
37.	Orji et al. [58]	2011	Rivers	SS	Neonate	Cerebrospinal fluid and blood	Invasive	145	21	14	NR	NR
38.	Patrick et al. [59]	2024	Bayelsa	SS		Swabs sample	Colonizing	185	11	6	NR	NR
39.	Rotimi et al. [60]	1985	Lagos	SW	Neonates	Umbilical cord swab	Colonizing	23	6	26	NR	NR
40.	Samuel et al. [61]	2019	Plateau	NC	Pregnant and non-pregnant women	Vaginal and rectal swabs	Colonizing	300	19	6	Ia (8), III (6), V (3), and II (2)	NR
41.	Uhiara [62]	1993	Kaduna	NW	Pregnant women and neonates	Vaginal and perineal swabs	Colonizing	200	27	14	Not clear	NR
42.	Wonodi [63]	2020	Rivers	SS	Children	Throat swab	Colonizing	456	23	5	NR	NR
43.	Yerumoh et al. [64]	2017	Edo	SS	Pregnant Women	Vaginal and rectal swabs	Colonizing	234	23	10	NR	NR

Keys: NC = North Central; NE = North East; NW = North West; SE = South East; SS = South South; SW = South West; NT = Non-typeable; NR = Not reported.

### 2.9. Reporting Bias Assessment

Publication bias was assessed visually using funnel plots, tables, and formally with trim-and-fill analysis and Egger’s regression tests; a *p*-value ≤ 0.05 was considered evidence of small-study effects.

## 3. Results

### 3.1. Study Selection and Study Characteristics

Of the 426 records retrieved, 43 met the inclusion criteria after screening (Figure 1). The characteristics of these forty-two studies are summarized in Table 1.

### 3.2. Prevalence of Group B Streptococcus Colonization in Nigeria

**Figure 2:**
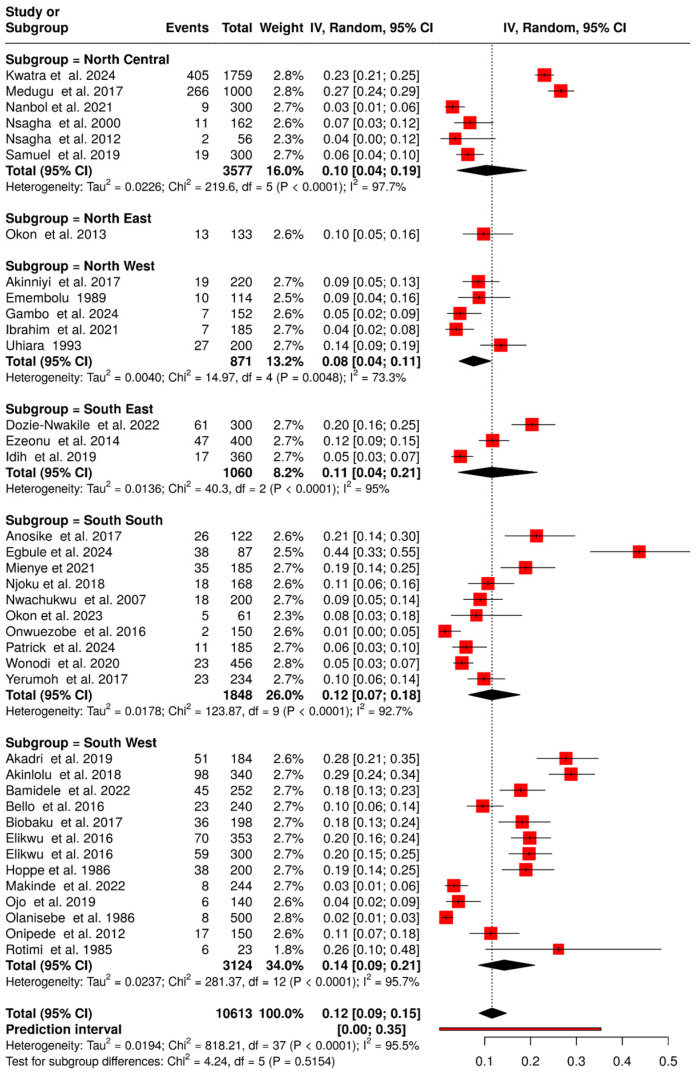
Forest plot of GBS colonization prevalence in Nigeria and geopolitical zones (1984–2024). The pooled prevalence was estimated using random-effects models [12,17,18,21,22,23,24,29,30,31,32,33,35,36,37,38,40,41,42,43,44,45,46,47,48,49,50,53,54,55,56,57,59,60,61,62,63,64]. Each black horizontal line represents a study’s 95% confidence interval, with red squares indicating point estimates and weights. Black diamonds denote comparisons, while dotted or dashed lines show subgroup analyses. The red horizontal line represents the prediction interval.

**Figure 3:**
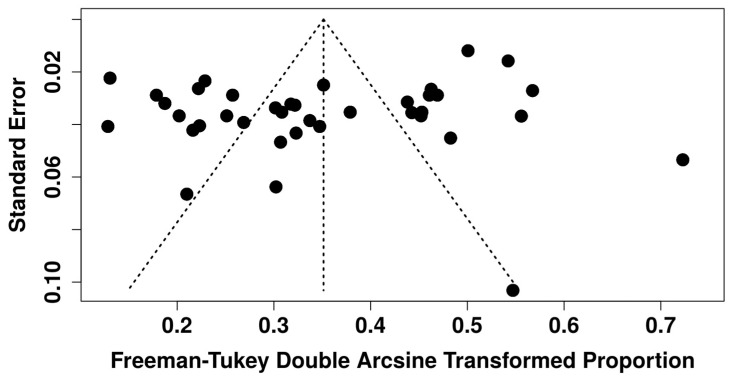
The funnel plot of studies conducted on Group B *Streptococcus* colonization rates in Nigeria between 1984 and 2024 demonstrates an asymmetrical distribution.

**Figure 4:**
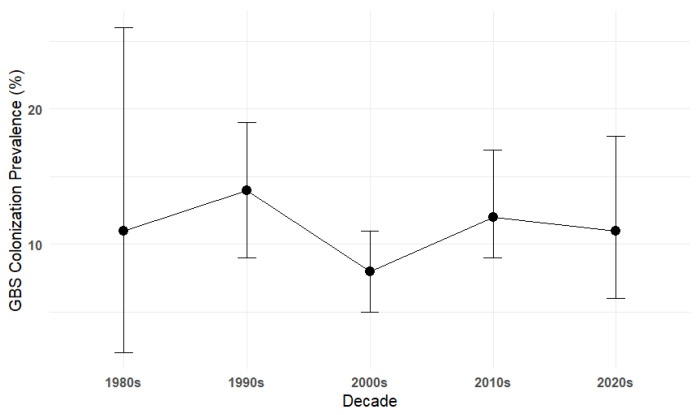
Colonization prevalence of Group B *Streptococcus* by decade with 95% confidence intervals. Black circles show the estimated GBS prevalence per decade, with vertical black lines represent 95% confidence intervals.

Of the 43 included studies, 38 reported GBS colonization, resulting in 1584 GBS-positive cases among 10,613 individuals, which translates to an overall prevalence of 12.0% (95% CI: 9.0–15.0%) (Figure 2). Evidence of publication bias was suggested by funnel plot asymmetry and Egger’s test (intercept: −4.22, *p* = 0.034) (Figure 3, Supplementary Table S5). To further assess the impact of potential publication bias, a trim-and-fill analysis was conducted under a random-effects model. No potentially missing studies were imputed (k_0_ = 0), and the adjusted pooled prevalence remained unchanged, indicating that the overall estimate was robust to the influence of publication bias. In addition, sensitivity analysis excluding two large studies [12,17] and one small study [60] yielded a prevalence of 11.0% (95% CI: 8.0–14.0%), while heterogeneity remained high (I^2^ = 93.6%) and no evidence of publication bias (Egger’s intercept: 2.47, *p* = 0.401), confirming the robustness of estimates. To explore sources of heterogeneity, a meta-regression was conducted using study years (in decades) as a moderator, with the 1980s as the reference category. The baseline prevalence in the 1980s was 11.0% (95% CI: 2.0–22.0%). No statistically significant differences were observed across subsequent decades (1990s–2020s), as reflected by overlapping confidence intervals, indicating the absence of a clear temporal trend (Figure 4). Substantial residual heterogeneity remained after adjustment.

Prevalence was higher in the South (13.0% [95% CI: 9.0–17.0%]) than in the North (9.0% [95% CI: 5.0–15.0%]). Across geopolitical zones, the South West recorded the highest prevalence (14.0% [95% CI: 9.0–21.0%]; 13 studies [18,21,22,29,31,32,37,38,43,50,55,56,60]), followed by the South South (12.0% [95% CI: 7.0–18.0%]; 10 studies [23,33,36,47,49,54,57,59,63,64]). The South East showed 11.0% (95% CI: 4.0–21.0%; 3 studies [33,45,63]), the North Central 10.0% (95% CI: 4.0–19.0%; 6 studies [12,17,24,46,48,61], and the North East 10.0% (95% CI: 5.0–16.0%; 1 study [53]). The lowest prevalence was in the North West (8.0% [95% CI: 4.0–11.0%]; 5 studies [30,40,42,44,62]).

**Figure 5:**
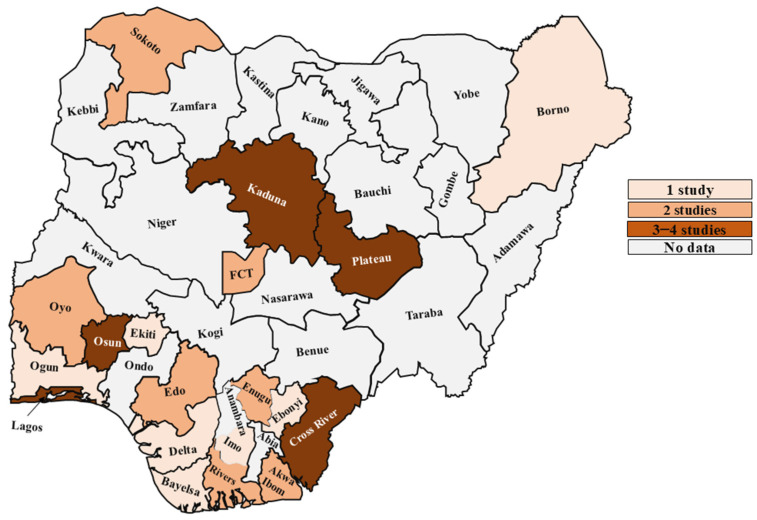
Distribution of studies across Nigerian states from 1984 to 2024, highlighting the range of data available per state.

No data were available for 18 states, including Ondo, Abia, Anambra, Ebonyi, Benue, Kogi, Kwara, Nasarawa, Adamawa, Bauchi, Gombe, Taraba, Yobe, Jigawa, Kano, Katsina, Kebbi, and Zamfara (Figure 5).

### 3.3. Prevalence of Group B Streptococcus Colonization in the Neonate

**Figure 6:**
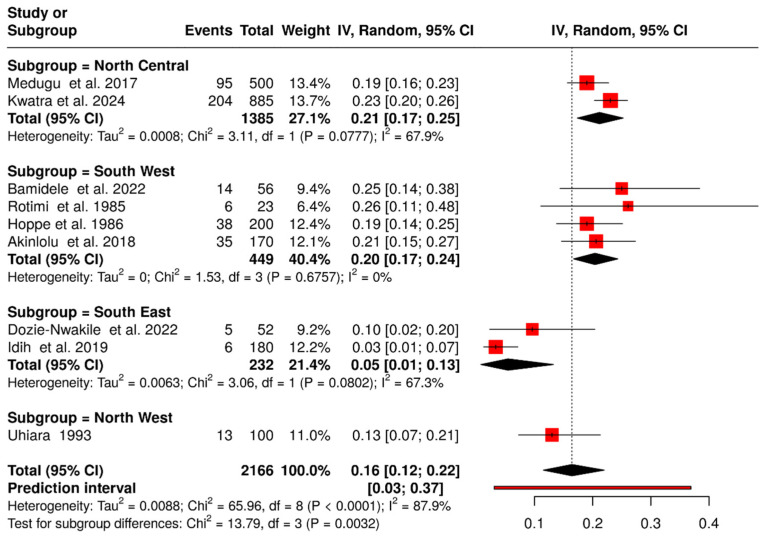
Forest plot of Neonatal GBS colonization prevalence in Nigeria and per geographical zones (1984–2024). The pooled prevalence was estimated using random-effects models [12,17,29,31,35,43,45,60,62]. Each black horizontal line represents a study’s 95% confidence interval, with red squares indicating point estimates and weights. Black diamonds denote comparisons, while dotted or dashed lines show subgroup analyses. The red horizontal line represents the prediction interval.

Nine of the 38 included studies reported on GBS colonization in neonates, involving a total of 2166 neonates across nine states of Nigeria [12,17,29,31,35,43,45,60,62]. Among these, 416 cases of GBS were identified, yielding a pooled prevalence of 16.0% (95% CI: 12.0–22.0%) (Figure 6).

Regionally, the northern part of the country reported the highest number of neonatal GBS cases, with 312 cases out of 1485 neonates, corresponding to a prevalence of 19.0% (95% CI: 15.0–24.0%) [12,17,62]. In contrast, the southern region reported 104 cases out of 681 neonates, with a prevalence of 16.0% (95% CI: 8.0–25.0%) [18,29,31,43,45].

### 3.4. Prevalence of Group B Streptococcus Colonization in Pregnant Women

**Figure 7:**
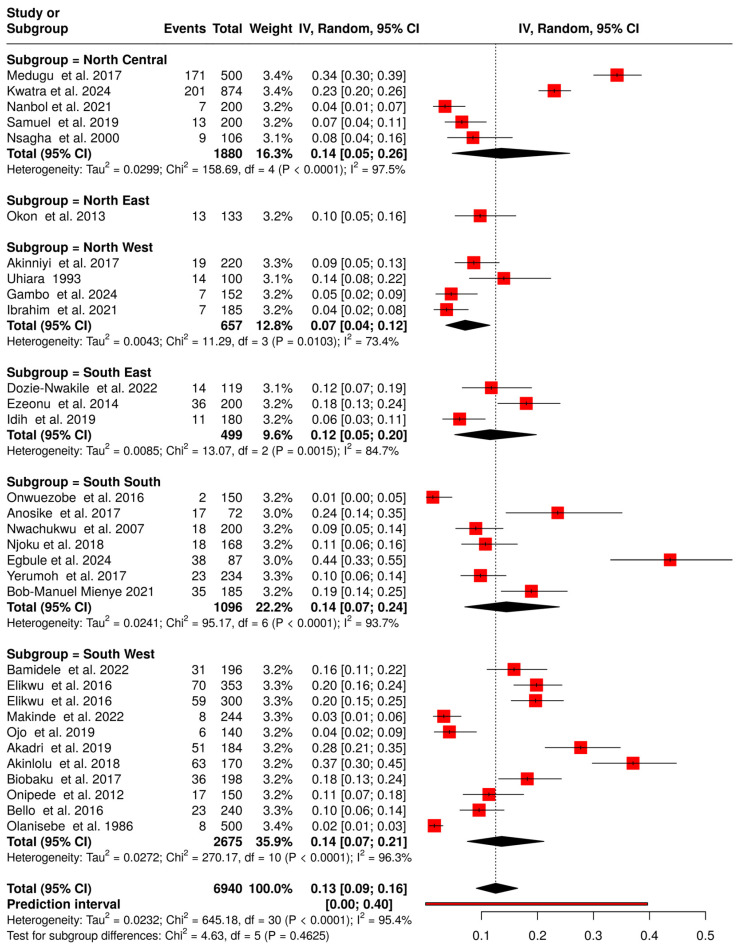
Forest plot of Maternal GBS colonization prevalence in Nigeria and geopolitical zones (1984–2024). The pooled prevalence was estimated using random-effects models [12,17,18,21,22,23,29,30,31,32,33,35,36,37,38,41,42,44,45,46,47,48,49,50,53,55,56,57,61,62,64]. Each black horizontal line represents a study’s 95% confidence interval, with red squares indicating point estimates and weights. Black diamonds denote comparisons, while dotted or dashed lines show subgroup analyses. The red horizontal line represents the prediction interval.

Thirty-one of the 38 included articles reported maternal colonization in Nigeria, involving a total of 6,940 women, of whom 1,045 were colonized, resulting in an overall prevalence of 13% (95% CI: 9.0–16.0%) (Figure 7). Prevalence was higher in the southern region of Nigeria (14.0% [95% CI: 9.0–18.0%]) compared to the northern region (11.0% [95% CI: 5.0–18.0%]).

Regional analysis showed that prevalence was highest in the North Central (14.0% [95% CI: 5.0–26.0%]; 5 studies [12,17,46,48,61] and lowest in the North West (7.0% [95% CI: 4.0–12.0%]; 4 studies [30,42,44,62]). The North East (10.0% [95% CI: 5.0–16.0%]; 1 study [53]) had intermediate prevalence. In the southern regions, South South (14.0% [95% CI: 7.0–24.0%]; 7 studies [23,33,36,47,49,57,64]), South East (12.0% [95% CI: 5.0–20.0%]; 3 studies [35,41,45]), and South West (14.0% [95% CI: 7.0–21.0%]; 11 studies showed similar prevalence across regions [18,21,22,29,31,32,37,38,50,55,56].

### 3.5. Prevalence of Maternal Group B Streptococcus Colonization in HIV Pregnant Women

Three studies assessed GBS colonization among HIV-positive pregnant women [18,32,47]. The individual studies reported prevalence estimates of 3.0% [18], 15.0% [47], and 19.0% [32], respectively, reflecting variability across study populations and settings. When the data were pooled, the overall prevalence of GBS colonization in this subgroup was estimated at 11.0% (95% CI: 3.0–25.0%), suggesting that HIV-positive women may have a moderate burden of GBS colonization. However, the wide confidence interval indicates uncertainty in the estimate and highlights the need for further studies with larger sample sizes to provide more precise prevalence data.

### 3.6. Impact of HIV on Maternal Group B Streptococcus Colonization

Three studies assessed maternal GBS colonization in HIV-positive and HIV-negative pregnant women [18,32,47]. The odds ratios from individual studies ranged from 1.00 to 2.89. A pooled Mantel–Haenszel meta-analysis showed no statistically significant association between HIV status and GBS colonization (OR = 1.47, 95% CI: 0.85–2.54, *p* = 0.17) (Table S5).

### 3.7. Prevalence of Group B Streptococcus Colonization in Non-Pregnant Individuals

**Figure 8:**
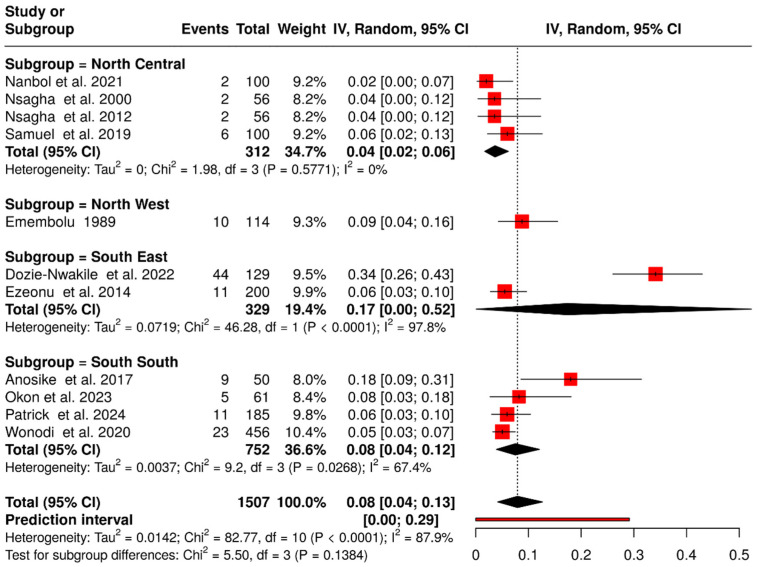
Forest plot of prevalence of colonization of GBS in non-pregnant, non-neonate in Nigeria and per geographical zones (1984–2024). The pooled prevalence was estimated using random-effects models [23,24,35,40,41,46,48,53,59,61,63]. Each black horizontal line represents a study’s 95% confidence interval, with red squares indicating point estimates and weights. Black diamonds denote comparisons, while dotted or dashed lines show subgroup analyses. The red horizontal line represents the prediction interval.

In addition to neonates and pregnant women, 11 studies reported GBS colonization among non-pregnant women, men, and school children [23,24,35,40,41,46,48,54,59,61,63], with a pooled prevalence of 8.0% (95% CI: 4.0–13.0%) (Figure 8). Of these, five studies were conducted in the northern region, involving 426 individuals, among whom 22 GBS isolates were identified, corresponding to a prevalence of 5.0% (95% CI: 3.0–8.0%) [24,40,46,48,61]. In contrast, studies from the southern region reported 103 GBS isolates out of 1081 individuals, yielding a prevalence of 11.0% (95% CI: 4.0–20.0%) [23,35,41,54,59,63].

### 3.8. Prevalence of Invasive Group B Streptococcus Cases

**Figure 9:**
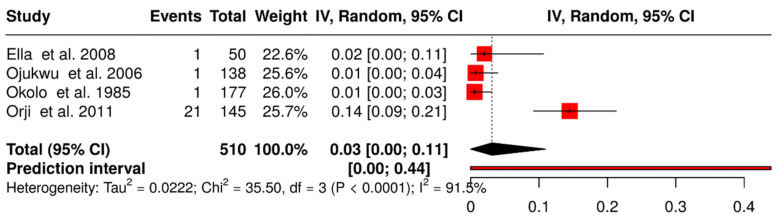
Forest plot of prevalence of invasive GBS infection in Nigeria (1984–2024). The pooled prevalence was estimated using random-effects models [39,51,52,58]. Each black horizontal line represents a study’s 95% confidence interval, with red squares indicating point estimates and weights. Black diamonds denote comparisons, while dotted or dashed lines show subgroup analyses. The red horizontal line represents the prediction interval.

Four studies out of the 43 included studies in this review reported invasive GBS in Nigeria, with an overall pooled prevalence of 3.0% (95% CI: 0.0–11.0%) (Figure 9) [39,51,52,58]. Interestingly, all occurred in neonates. In Kaduna, GBS was isolated from 1 of 50 neonates with sepsis (7.69%) [39]. Ojukwu et al. [51] and Okolo and Omene [52] each identified one case of neonatal septicaemia, with overall incidences of 7.98 and 5.6 per 1000 live births, respectively. In Rivers State, GBS was detected in 21 of 145 infant blood or cerebrospinal fluid samples (14.5%) [58].

### 3.9. Capsular Serotype Distribution of Group B Streptococcus in Nigeria

Of the 43 included studies, seven reported capsular serotype distribution [12,17,33,34,37,43,61]. Of these, four studies were included in the analysis, while three were excluded: two (Hoppe et al. [43] and Kwatra et al. [12]) had unclear serotype data, and one (Bob-Manuel et al. [34]) performed serotyping on isolates that had already been classified. Across the four studies, 356 GBS isolates were serotyped. The most common identified serotype was serotype V (137, 38.5%). Others include serotype II (78, 21.9%), serotype Ia (63, 17.7%), serotype III (53, 14.9%), serotype Ib (10, 2.8%), serotype IV (7, 2.0%), serotype VI (1, 0.3%), and non-typeable (NT) isolates (7, 2.0%).

Individual studies showed variable distributions. Samuel et al. [61] observed serotype Ia (42.1%), III (31.6%), V (15.8%), and II (10.5%). Elikwu et al. [37] reported serotype Ia (23.9%) predominating in maternal isolates and serotype II (71.4%) in neonates. Bob-Manuel et al. [33] identified serotype V (32.6%) and serotype Ia (21.7%) as most common among pregnant women, while Medugu et al. [17] reported serotype V (45.7%), II (24.5%), Ia (13.9%), III (11.8%), NT (2.9%), and IV (1.2%).

### 3.10. Sequence Type Distribution of Group B Streptococcus

Two studies reported MLST data in Nigeria [17,34], identifying 14 STs. The combined STs from both studies included ST19 (*N* = 18, 26.5%), ST182 (*N* = 10, 14.7%), ST28 (n = 6, 8.8%), ST1, ST24, and ST486 (*N* = 5 each, 7.4%), ST17 and ST23 (*N* = 4 each, 5.9%), ST2 (*N* = 3, 4.4%), ST8, ST26, and ST1336 (*N* = 2 each, 2.9%), and ST196 and ST762 (*N* = 1 each, 1.5%).

Individually, Medugu et al. in 2017 reported STs included ST19 (*N* = 9, 25.7%), ST182 (*N* = 7, 20.0%), ST1 (*N* = 4, 11.4%), ST28, ST17, and ST23 (*N* = 3 each, 8.6%), and ST2, ST196, ST8, ST26, ST24, and ST762 (*N* = 1 each, 2.9%) [17]. Similarly, Bob-Manuel et al. (2021) reported 12 STs: ST19 (*N* = 9, 27.3%), ST486 (*N* = 5, 15.2%), ST24 (*N* = 4, 12.1%), ST28 and ST182 (*N* = 3 each, 9.1%), ST2 and ST1336 (*N* = 2 each, 6.1%), and ST1, ST8, ST17, ST23, and ST26 (*N* = 1 each, 3.0%) [34].

## 4. Discussion

GBS is a significant pathogen of public health importance, widely recognized as both a neonatal and maternal pathogen. Globally, colonization varies by geography and socioeconomic context [2,6,7,16,65]. Our meta-analysis found an overall prevalence of 12.0% (95% CI: 9.0–15.0%) in Nigeria, with neonatal colonization at 16.0% (95% CI: 12.0–22.0%), maternal colonization at 13.0% (95% CI: 9.0–16.0%), and 8.0% (95% CI: 4.0–13.0%) in non-pregnant populations. These figures highlight Nigeria’s contribution to the global GBS burden and illustrate regional variation that requires contextual interpretation.

The 16.0% neonatal colonization rate is concerning compared with global and African estimates [2,66,67,68]. A higher prevalence in northern Nigeria (19.0%) compared to the south (16.0%), this may suggest differences in the dynamics of vertical transmission. Invasive neonatal disease was detected in 3.0% (95% CI: 0.0–11.0%), consistent with global trends [2,66]. Although neonatal GBS is under-documented in Nigeria, our findings support its role in early-onset sepsis [45,65,69]. Africa bears the highest global incidence (1.12 per 1000 live births vs. 0.49 per 1000 globally) [67], and Nigerian data align with this pattern [12,17,39,51,52,58,67].

The maternal prevalence in Nigeria was 13.0%, which is lower than the global estimate of 18% (95% CI: 17–19%) [6] and lower than the average rates in other African countries (16–22%) [7,14,15,65]. Methodological variation, such as swab site, timing, culture techniques, and sample size, likely contributed to this lower pooled prevalence [7,62]. Regionally, rates were higher in the south (14%) compared to the north (11%), mirroring the broader continental heterogeneity [9,16,70,71]. GBS colonization in HIV-positive pregnant women has been examined in multiple studies from different countries [71,72,73]. Among HIV-positive pregnant women, prevalence was 11.0% (95% CI: 3.0–25.0%) with no significant association between HIV and colonization (OR = 1.47, 95% CI: 0.66–3.24). This aligns with findings from Malawi (19.4% vs. 21.7%), Brazil (19.8% vs. 14.15%), Belgium (29.6% vs. 24.2%), and the United States (41.3% vs. 30.6%) [74,75,76,77], although data from Tanzania suggest an increased risk (63.1% vs. 18.9%) [73]. Given the wide confidence intervals and few Nigerian studies, larger, prospective cohorts are needed to clarify this relationship [18,32,47].

Beyond neonates and pregnant women, GBS is also an important pathogen on the broader population [4,8,78,79,80]. Globally, it has been reported in different forms of infection, including throat infections in school-aged children and young adults [63,81,82], urinary tract infections in men and women, and even infertility [40,83,84,85] and sepsis and cellulitis [79,80]. Our pooled prevalence among this population was 8.0%, comparable to global estimates (9–32%) [4,78,86,87,88]. Nigerian data fall within the lower range of global values, although regional differences were evident, with a higher prevalence in the south (11%) compared to the north (5%).

The serotype distribution in Nigeria reflects the global epidemiology. Across the five studies that conducted serotyping, serotype V was predominant [12,17,33,37,43]. These, along with serotypes Ia, Ib, II, and III, account for over 90% of invasive cases globally and are targets of candidate maternal vaccines [7,14,67]. There is considerable variation in GBS serotype distribution across countries. Kwatra et al. reported serotype Ia as the most prevalent in Bangladesh, India, Ethiopia, Kenya, and South Africa, serotype V in Mali, and serotype III in Mozambique [12]. In Southeast Asian countries, Muthanna and colleagues identified serotype III as the predominant serotype, followed by serotypes V, II, VI, and Ia [6]. In Europe, North America, and Southern Africa, serotype IV was more commonly reported, whereas serotype V predominated in West Africa [11].

Like other African countries, there are limited GBS MLST data in Nigeria. Two studies, Medugu et al. and Bob-Manuel et al., conducted MLST, reporting 14 GBS STs with ST19 (26.5%), ST182 (14.7%), and ST28 (8.8%) predominated [17,34]. In Kenya, ST17 and ST23 were the most reported, followed by ST10 and ST1. In Senegal, ST1, ST26, and ST28 were the most common [89,90]. Outside Africa, studies from Malaysia and Thailand indicate ST1 as the most prevalent STs, followed by ST17 and ST283 [6,91,92]. A Canadian study similarly reported ST1 as the most common, followed by ST23 [93]. Unlike regions where hypervirulent ST17 dominates, Medugu et al. and Bob-Manuel et al. reported low (5.9%). The availability of only two MLST studies in Nigeria highlights the need for expanded molecular epidemiology. Without additional data, it is difficult to track the circulation and evolution of GBS strains or assess their potential virulence in Nigeria. Expanded surveillance, including MLST or whole-genome sequencing, would provide critical information on STs, clonal complexes and virulence factors, helping to guide vaccine implementation and public health strategies in Nigeria [11,17,94].

Nigeria’s 13% maternal colonization prevalence suggests opportunities for prevention. In high-income countries, universal screening and IAP have reduced EOD [1,5]. While universal screening may be impractical in Nigeria, risk-based or culture-limited approaches could be introduced in tertiary centers [17,95]. The cost of implementing screening (₦300,000/500 women) is substantially lower than treating neonatal sepsis or its sequelae [17]. Standardized rectovaginal swabs, improved culture capacity, and national perinatal pathogen surveillance are essential. Antenatal care should be updated to clarify and include IAP. Preparations for maternal GBS vaccines should include monitoring of serotype and lineage, particularly in under-studied northern and rural states.

This study addresses an important question and represents the first systematic review and meta-analysis of GBS in Nigeria. By pooling data from pregnant women, neonates, non-pregnant adults, and HIV-positive populations, it provides a broad national overview. Methodological rigor, adherence to PRISMA, and sensitivity analyses further strengthen the reliability of findings. However, high heterogeneity (I^2^ > 90%) across studies, reflecting differences in methods, swab sites, and culture techniques, limits comparability. Study quality was assessed using the JBI checklists; however, a formal GRADE assessment was not conducted. Similarly, differences in rates may reflect variation in surveillance, screening, and research coverage rather than true biological differences, as only 19 of 37 states contributed data, leaving significant geographical gaps. Most data came from urban hospital settings, which may have led to an underestimation of rural prevalence.

Overall, the findings suggest that GBS colonization and disease are underrecognized in Nigeria, and the prevalence should be interpreted with caution, as it does not reflect uniform national coverage. They emphasize the need to enhance standardized GBS screening and diagnostic capabilities in maternal and neonatal care, supported by national guidelines and coordinated surveillance, to address existing geographical disparities. Future research should focus on multi-state, population-based studies and greater inclusion of rural populations to better inform prevention and control strategies.

## 5. Conclusions

This systematic review synthesizes four decades of GBS data in Nigeria, revealing an overall prevalence of 12%, higher in neonates (16%) than in pregnant women (13%) or other populations (8%). Findings align with global trends in serotypes and STs although hypervirulent ST17 appears to be less prevalent. Expanding surveillance, introducing feasible prevention measures, and preparing for maternal vaccines are vital steps to address neonatal invasive infections and strengthen maternal–child health outcomes.

## Data Availability

The original contributions presented in this study are included in the article/Supplementary Material. Further inquiries can be directed to the corresponding authors.

## Supplementary Materials

The following supporting information can be downloaded at: https://www.mdpi.com/article/10.3390/medsci14010093/s1, Table S1: PRISMA Checklist; Table S2: Search Queries; Table S3: Excluded Articles with Reasons [69,96,97,98,99,100,101,102,103,104,105,106,107,108,109,110,111,112,113,114,115,116]; Table S4: Quality Assessment of Included Studies; Table S5: Publication Bias Assessment Result; Table S6: Odds ratios (OR) of GBS colonization by maternal HIV status in Nigeria.

## Abbreviations

The following abbreviations are used in this manuscript:

GBS	Group B *Streptococcus*
PROSPERO	International Prospective Register of Systematic Reviews
PRISMA	Preferred Reporting Items for Systematic reviews and Meta-Analyses
MLST	Multilocus Sequence Typing
ST	Sequence Type
HIV	Human Immunodeficiency Virus
IAP	Intrapartum Antibiotic Prophylaxis
EOD	Early-Onset Disease
OR	Odd Ratio
CI	Confidence Interval
