# Supplementary material for: Prevalence of Group B Streptococcus Colonization and Invasive Infection in Nigeria: A Systematic Review and Meta-Analysis
Source: Med Sci (Basel). 2026 Feb 15;14(1):93. doi: 10.3390/medsci14010093 (PMC12921764; doi:10.3390/medsci14010093)
Supplement: Supplementary file 1 [file medsci-14-00093-s001.zip › Supplementary S2 - S6.pdf]

Supplementary Table 2: Search Queries

Database	#	Search Query	Result (No. of Articles)
Scopus	1	"Streptococcus agalactiae" OR "Group B Streptococcus" OR "Group B streptococci" OR "Group B streptococcal"	21863
	2	Nigeria	136584
	1 AND 2	(TITLE-ABS-KEY ("Group B streptococcus" OR "Streptococcus agalactiae" OR "Group B streptococcal") AND TITLE-ABS-KEY (Nigeria))	44
PubMed	1	(TITLE-ABS-KEY ("Streptococcus agalactiae" OR "Group B Streptococcus" OR "Group B streptococci" OR "Group B streptococcal") AND TITLE-ABS-KEY (Nigeria))	13592
	2	Nigeria	86647
	1 AND 2	(("Streptococcus agalactiae"[Mesh] OR "Streptococcus agalactiae"[tiab] OR "Group B Streptococcus"[tiab] OR "Group B streptococci"[tiab] OR "Group B streptococcal"[tiab])) AND (Nigeria)	38
ScienceDirect	1 AND 2	"Streptococcus agalactiae" OR "Group B Streptococcus" OR "Group B streptococci" OR "Group B streptococcal" AND Nigeria	260
Dimension	1	"Streptococcus agalactiae" OR "Group B Streptococcus" OR "Group B streptococci" OR "Group B streptococcal"	131466
	2	Nigeria	278820
	1 AND 2	"Streptococcus agalactiae" OR "Group B Streptococcus" OR "Group B streptococci" OR "Group B streptococcal" AND Nigeria	12
Web of Science	1	Streptococcus agalactiae OR "Group B Streptococcus" OR "Group B streptococci" OR "Group B streptococcal"	12471
	2	Nigeria	80022
	1 AND 2	Streptococcus agalactiae OR "Group B Streptococcus" OR "Group B streptococci" OR "Group B streptococcal" AND Nigeria	20
AJOL and Google Search		Streptococcus agalactiae OR "Group B Streptococcus" OR "Group B streptococci" OR "Group B streptococcal" AND Nigeria	10
Goggle Scholar		"Streptococcus agalactiae" OR "Group B Streptococcus" OR "Group B streptococci" OR "Group B streptococcal" AND Nigeria	42

Supplementary table 3: Excluded Articles with Reasons

S/N	Authors	Year	Title	Reason for Exclusion
1.	Onile [96]	1984	Group B streptococcal neonatal and infant infections in Nigeria: a review and update.	Case report
2.	Danmallam et al. [97]	2019	Study on prevalence, clinical presentation, and associated bacterial pathogens of goat mastitis in Bauchi, Plateau, and Edo States, Nigeria	GBS in Goat Mastitis
3.	Airede et al. [98]	2008	Neonatal bacterial meningitis and dexamethasone adjunctive usage in Nigeria	GBS is not reported
4.	Ako-Nai et al. [99]	1999	The bacteriology of neonatal septicaemia in Ile-Ife, Nigeria	GBS is not reported
5.	Obadare et al. [100]	2023	Clinical and bacteriological profiles of neonatal sepsis in a tertiary hospital, south-western Nigeria	GBS is not reported
6.	Airede [101}	1993	Neonatal bacterial meningitis in the middle belt of Nigeria.	GBS is not reported
7.	Longe et al. [102]	1984	Neonatal meningitis in Nigerian infants.	GBS is not reported
8.	Lawal and Ogugbemi [103]	1989	Antibiogram of Lagos strains of beta-haemolytic streptococci.	No access
9.	Nwachukwu [104]	2024	Antibacterial resistance patterns of Group B Streptococcus infections in pregnant women attending St John's Hospital, Owerri, Nigeria	No access
10.	Nwankwu and Njoku-Obi [105]	1990	Serogroups of beta-haemolytic streptococci isolated from clinical specimens in parts of eastern Nigeria.	No access
11.	Omololu-Aso [106]	2018	Group B Streptococcus in pregnancy and neonatal colonization at primary health care institutions in Nigeria	No access
12.	Nwadioha et al. [107]	2013	Bacterial isolates from cerebrospinal fluid of children with suspected acute meningitis in a Nigerian tertiary hospital.	Patient not clear
13.	Onile [108]	1980	Group B streptococcal carriage in Nigeria	Not study year
14.	Onile et al [109]	1980	Neonatal Septicemia Resulting from Group B Streptococci: A Case Report	Not study year
15.	Manning et al. [110]	2003	Correlates of antibiotic-resistant group B streptococcus isolated from pregnant women	Outside Nigeria
16.	Mueller et al. [111]	2014	Intrapartum detection of Group B streptococci colonization by rapid PCR-test on labor ward	Outside Nigeria
17.	Elikwu et al. [112]	2014	Antibiotic susceptibility profiles of group B streptococci (GBS) isolates from pregnant mothers in a tertiary institution in Nigeria	Previous GBS isolates
18.	Omololu-Aso et al. [113]	2017	A Case study of Group B Streptococcus Associated with Women in Antepartum Period and their Neonates, Ile Ife South-western Nigeria	Sample and result not clear
19.	Adewumi et al. [114]	2017	Microbiological pattern in preterm prelabour rupture of the fetal membranes in South-Western Nigeria	Streptococcus unclassified
20.	Onalo et al. [115]	2011	Rate of isolation of streptococcus species from children with bacterial infections: an indication for introduction of streptococcal vaccines	Streptococcus unclassified

21.	Medugu et al. [116]	2018	Aetiology of neonatal sepsis in Nigeria, and relevance of group b streptococcus: A systematic review	Review
22.	Le Bris et al. [117]	2024	Investigating Group B Streptococcus (GBS) genetic factors associated with vertical transmission and invasive neonatal disease in Nigeria	Evolutional studies

Supplementary Table 4: Quality assessment of included studies

Criteria selected to determine the quality of studies.

A. Is the study's objective stated explicitly?

B. Clearly defined criteria for including and excluding participants

C. Are the sample collection clearly described?

D. Used a standardized procedure for bacterial identification

E. Are the primary outcomes reported in detail?

F. Are the participants' characteristics adequately described?

G. Are the key results of the study presented clearly?

H. Do the participants invited to join the study reflect the broader source population?

I. Were the statistical methods applied to the main outcomes suitable?

J. Were the outcome measures used both valid and reliable?

S/N	Authors	Year	A	B	C	D	E	F	G	H	I	J	Score
1	Akadri et al. [21]	2019	Yes	Yes	Yes	Yes	Yes	Yes	Yes	Yes	Yes	Yes	10
2	Akinlolu et al. [29]	2018	Yes	Yes	Yes	Yes	Yes	Yes	Yes	Yes	Yes	No	9
3	Akinniyi et al. [30]	2017	Yes	Yes	Yes	Yes	Yes	Yes	Yes	Yes	Yes	Yes	10
4	Anosike et al. [23]	2017	Yes	Yes	Yes	Yes	Yes	Yes	Yes	Yes	Yes	Yes	10
5	Bamidele et al. [31]	2022	Yes	Yes	Yes	Yes	Yes	Yes	Yes	Yes	Yes	Yes	10
6	Bello et al. [22]	2016	Yes	Yes	Yes	Yes	Yes	Yes	Yes	Yes	Yes	Yes	10
7	Biobaku et al. [32]	2017	Yes	Yes	Yes	Yes	Yes	Yes	Yes	Yes	Yes	Yes	10
8	Bob-Manuel et al. [33]	2021	Yes	Yes	Yes	Yes	Yes	Yes	Yes	Yes	Yes	Yes	10
9	Dozie-Nwakile et al. [35]	2022	Yes	Yes	Yes	Yes	Yes	Yes	Yes	Yes	Yes	Yes	10
10	Egbule et al. [36]	2024	Yes	Yes	Yes	Yes	Yes	Yes	Yes	Yes	Yes	Yes	10
11	Elikwu et al. [37]	2016	Yes	Yes	Yes	Yes	Yes	Yes	Yes	Yes	Yes	Yes	10
12	Elikwu et al. [38]	2016	Yes	Yes	Yes	Yes	Yes	Yes	Yes	Yes	Yes	No	9
13	Ella et al. [39]	2008	Yes	Yes	Yes	Yes	Yes	Yes	Yes	Yes	Yes	Yes	10
14	Emembolu [40]	1989	Yes	Yes	Yes	Yes	Yes	Yes	No	Yes	No	Yes	8
15	Ezeonu and Agbo [41]	2014	Yes	Yes	Yes	Yes	Yes	Yes	Yes	Yes	Yes	Yes	10
16	Gambo et al. [42]	2024	Yes	Yes	Yes	Yes	Yes	Yes	Yes	Yes	Yes	Yes	10
17	Hoppe et al. [43]	1986	Yes	Yes	Yes	Yes	Yes	Yes	No	Yes	Yes	Yes	9
18	Ibrahim et al. [44]	2021	Yes	Yes	Yes	Yes	Yes	Yes	No	Yes	Yes	Yes	9
19	Idih et al. [45]	2019	Yes	Yes	Yes	Yes	Yes	Yes	Yes	Yes	Yes	Yes	10
20	Kwatra et al. [12]	2024	Yes	Yes	Yes	Yes	Yes	Yes	Yes	Yes	Yes	Yes	10
21	Makinde et al. [18]	2022	Yes	Yes	Yes	Yes	Yes	Yes	Yes	Yes	Yes	Yes	10
22	Medugu et al. [17]	2017	Yes	Yes	Yes	Yes	Yes	Yes	Yes	Yes	Yes	Yes	10
23	Nanbol et al. [46]	2021	Yes	Yes	Yes	Yes	Yes	Yes	Yes	Yes	Yes	Yes	10
24	Njoku et al. [47]	2018	Yes	Yes	Yes	Yes	Yes	Yes	Yes	Yes	Yes	Yes	10
25	Nsagha et al. [48]	2000	Yes	Yes	No	Yes	Yes	No	No	Yes	Yes	Yes	70
26	Nsagha et al. [24]	2012	Yes	Yes	Yes	Yes	Yes	Yes	Yes	Yes	Yes	Yes	10
27	Nwachukwu et al. [49]	2007	Yes	Yes	Yes	Yes	Yes	Yes	Yes	Yes	Yes	Yes	10
28	Ojo et al. [50]	2019	Yes	Yes	Yes	Yes	Yes	Yes	Yes	Yes	Yes	No	9
29	Ojukwu et al. [51]	2006	Yes	Yes	Yes	Yes	Yes	Yes	Yes	Yes	Yes	Yes	10
30	Okolo et al. [52]	1985	Yes	Yes	Yes	Yes	Yes	Yes	Yes	Yes	Yes	Yes	10
31	Okon et al. [53]	2013	Yes	Yes	Yes	Yes	Yes	Yes	Yes	Yes	Yes	Yes	10

32	Okon et al. [54]	2023	Yes	Yes	Yes	Yes	Yes	Yes	Yes	Yes	Yes	Yes	10
33	Olanisebe and Adetosoye [55]	1986	Yes	Yes	Yes	Yes	Yes	Yes	Yes	Yes	Yes	Yes	10
34	Onipede et al. [56]	2012	Yes	Yes	Yes	Yes	Yes	Yes	Yes	Yes	Yes	Yes	10
35	Onwuezobe and Effiom [57]	2016	Yes	Yes	Yes	Yes	Yes	Yes	Yes	Yes	Yes	Yes	10
36	Orji et al. [58]	2011	Yes	Yes	Yes	Yes	Yes	Yes	Yes	Yes	Yes	No	9
37	Patrick et al. [59]	2024	Yes	Yes	Yes	NC	Yes	Yes	Yes	Yes	Yes	Yes	9
38	Rotimi et al. [60]	1985	Yes	Yes	Yes	Yes	Yes	Yes	No	Yes	Yes	No	8
39	Samuel et al. [61]	2019	Yes	Yes	Yes	Yes	Yes	Yes	Yes	Yes	Yes	Yes	10
40	Uhiara [62]	1993	Yes	Yes	Yes	Yes	Yes	Yes	Yes	Yes	No	No	8
41	Wonodi [63]	2020	Yes	Yes	Yes	Yes	Yes	Yes	Yes	Yes	Yes	Yes	10
42	Yerumoh et al. [64]	2017	Yes	Yes	Yes	Yes	Yes	Yes	Yes	Yes	Yes	Yes	10

Supplementary Table 5: Publication bias assessment using Egger's test and trim-and-fill analysis.

Item	Studies (k)	Pooled prevalence (%)	Egger's Intercept (p-value)	I^2 (%)	Trim-and-Fill: Imputed Studies (k_0)	Trim-and-Fill Pooled Prevalence (%)
Studies (38)	37	12 (9-15)	−4.22 (0.034)	95.5	0	12 (9-15)
GBS (3 excluded)	34	11 (8-14)	2.47 (0.401)	93.6	0	11. (8-14)

Supplementary Table 6. Maternal Group B Streptococcus Colonization by HIV Status in Nigeria

Study	PWHIV+ (n)	PWHIV+/ GBS+ (n)	PWNHIV+ (n)	PWNHIV+/ GBS+ (n)	(OR)	95% CI	p = value
Biobaku et al. [32] 2017	67	13	131	23	1.13	0.53-2.40	0.84
Makinde et al. [18] 2022	122	4	122	4	1.00	0.24-4.09	1.00
Njoku et al.[47] 2018	84	13	84	5	2.89	0.98-8.52	0.07
**Total**	**273**	**30**	**337**	**32**	-	-	
**Pooled (MH)**	**-**	**-**	**-**	**-**	**1.47**	**0.85–2.54**	**0.17**

HIV+ = HIV-positive pregnant women; HIV+/GBS+ = GBS-positive in HIV-positive women; HIV– = HIV-negative pregnant women; HIV–/GBS+ = GBS-positive in HIV-negative women; OR = odds ratio; 95% CI = 95% confidence interval; MH = Mantel–Haenszel pooled estimate
